# Circulating miR-130a-3p is elevated in patients with cerebral atherosclerosis and predicts 2-year risk of cerebrovascular events

**DOI:** 10.1186/s12883-022-02829-5

**Published:** 2022-08-22

**Authors:** Jialei Xu, Fengchao Gao

**Affiliations:** Department of Neurology, Liaocheng People’s Hospital, No. 45 Huashan Road, Liaocheng, 252000 Shandong China

**Keywords:** Atherosclerosis, Transient ischemic attack, Stroke, microRNA-130a-3p, Diagnosis, Cerebrovascular events, Prognosis

## Abstract

**Background:**

Cerebral atherosclerosis (AS) leads to high risk of cerebrovascular events. This study aims to evaluate the diagnostic performance of serum microRNA-130a-3p (miR-130a-3p) in cerebral AS patients, and construct a logistic risk model for 2-year cerebrovascular events on the basis of the prognostic potential of miR-130a-3p.

**Methods:**

Serum samples were collected from 74 cerebral AS patients and 62 control individuals, and miR-130a-3p expression was investigated using reverse transcription quantitative PCR. Risk factors related with cerebral AS were assessed using a logistic regression analysis, and the receiver operating characteristic analysis was performed to evaluate the diagnostic value of miR-130a-3p. The relationship between miR-130a-3p and cerebrovascular events was analyzed using a Kaplan–Meier method, and a logistic risk model was constructed for 2-year cerebrovascular events.

**Results:**

Cerebral AS patients had elevated serum miR-130a-3p compared with controls (*P* < 0.001). Serum miR-130a-3p had diagnostic value (AUC = 0.899), and could significantly improve the diagnostic accuracy of total cholesterol (TC) and low-density lipoprotein cholesterol (LDL-C) in cerebral AS patients (AUC = 0.992). High serum miR-130a-3p was independently related with high probability of cerebrovascular events (HR = 1.993, 95% CI = 1.205–2.897, *P* = 0.006), and a logistic risk model was constructed based on serum miR-130a-3p, hs-CRP, TC and LDL-C.

**Conclusion:**

All the findings indicated that high serum miR-130a-3p had diagnostic potential to screen cerebral AS, and predicted the probability of cerebrovascular events after AS. The logistic risk model based on miR-130a-3p may provide an efficient method to predict 2-year cerebrovascular events in AS patients.

## Introduction

Atherosclerosis (AS) is a major pathological basis of cerebral infarction, coronary heart diseases and peripheral vascular diseases, enhancing human mortality, especially in older age groups [1]. Cerebral AS is characterized by progressive lipid deposition, fibrous hyperplasia and inflammatory cell infiltration, and represents a kind of chronic diseases in brain supplying artery system [2]. The diagnosis and prognosis of cerebral AS need special attention because it can lead to serious adverse clinical outcomes, such as transient ischemic attack (TIA), stroke and even deaths [3]. The development and progression of AS are complex, and the dysfunction of various cells, for example, vascular smooth muscle cells and endothelial cells, plays pivotal roles in the pathogenesis of AS [4]. Thus, it is believed that the molecules related with AS-associated cell function may provide targets to develop the diagnosis, prognosis and treatment of cerebral AS.

MicroRNAs (miRNAs) are a group of highly conserved and functional endogenous non-coding small RNAs [5]. They regulate the expression of functional genes by directly targeting the 3'-untranslated region (UTR) of mRNAs, and thereby participate in the pathogenesis of various human diseases [6]. Some miRNAs have been demonstrated to be involved in the development and progression of AS, such as miR-217 [7] and miR-181b [8]. Several differentially expressed miRNAs, such as miR-126, miR-143 and miR-137, have been determined as candidate biomarkers for patients with cerebral AS [9, 10]. miR-130a-3p has been investigated in patients with carotid stenosis, and it was found to be elevated in stenosis progression [11]. In addition, miR-130a-3p has regulatory effects on the cell proliferation and migration of vascular smooth muscle cells, which are the critical pathomechanisms of AS [12, 13]. These previous findings indicated that there may be some relationship between miR-130a-3p and cerebral AS.

To understand the clinical role of miR-130a-3p, the expression of serum miR-130a-3p was examined in patients with cerebral AS, and its diagnostic performance to screen cerebral AS cases, and the relationship of miR-130a-3p with cerebrovascular events secondary by AS were evaluated. Additionally, a logistic risk model was constructed based on circulating miR-130a-3p, hoping to provide novel prediction methods for the occurrence of cerebrovascular events in cerebral AS patients.

## Materials and methods

### Study population and serum collection

Between 2018 and 2019, 136 individuals were enrolled from the Liaocheng People’s Hospital and grouped into cerebral AS group and control group based on the examination results of carotid artery, middle cerebral artery, anterior cerebral artery, basal artery and vertebral artery using cerebrovascular transcranial Doppler sonography, magnetic resonance angiography and arterial CT angiography. The cases with AS were grouped into cerebral AS group (*n* = 74). The rest 62 cases presented parts of clinical signs of AS, but were confirmed without cerebral AS after corresponding examinations were grouped into control group. None of the participants had history of stroke, severe heart diseases, dissection, severe infection diseases, liver and nephrosis diseases or any tumors. At the time of admission, venous blood was collected from the participants, and serum samples were isolated from the blood using centrifugation and then stored at -80℃ for further examinations. The study protocols were reviewed and approved by the Ethics Committee of Liaocheng People’s Hospital, and patient consent was obtained prior to the sample collection.

### Collection of analysis data

This study recorded the demographic data and clinical data of the participants, including age, gender, smoking, drinking, history of hypertension and diabetes, concentration of hypersensitivity C response protein (hs-CRP), total cholesterol (TC), triglyceride (TG), low-density lipoprotein cholesterol (LDL-C) and high-density lipoprotein cholesterol (HDL-C). Hypertension was defined as cases with resting systolic blood pressure ≥ 140 mmHg, and cases with fasting blood glucose ≥ 7.0 mM were considered as diabetes.

### Two-year follow up survey

Each cerebral AS patients were followed up for 2 years to investigate the incidence of cerebrovascular events. During the follow-up, patients were given anti-platelet drugs (aspirin or clopidogrel) and lipid-lowering drugs (atorvastatin calcium or rosuvastatin). The end points of the 2-year follow up survey were cerebrovascular events, including TIA, stroke and death, and the 2-year end date. The criterial from American Heart Association/American Stroke Association was used to define TIA and stroke [14]. The follow-up survey was achieved by telephone or outpatient consultation.

### Extraction of total RNA

TRIzol Reagent (Invitrogen, Carlsbad, CA, USA) was used to extract total RNA from the serum samples following the manufacturer’s instruction, and a NanoDrop 2000 (Thermo Fisher, Scientific, Inc.) was adopted to measure the purity of the RNA. Only the RNA with a ratio of OD260/OD280 closed to 2.0 was used for subsequent experiments.

### Reverse transcription quantitative PCR (RT-qPCR)

Reverse transcription was firstly performed to synthesize cDNA from RNA using a PrimeScript RT reagent kit (TaKaRa, Tokyo, Japan) according to the instruction of manufacturer. Then, the cDNA was used as template for qPCR, which was carried our using SYBR Green I Master Mix kit (Invitrogen, CA, USA) and ABI 7500 QRT-PCR System (Applied Biosystems, Foster City, CA, USA). The PCR conditions were as follows: initial denaturation at 95℃ for 5 min, followed by 40 cycles of 95℃ for 30 s, 59℃ for 15 s, 72℃ for 20 s. U6 was used as an internal control, and the relative expression of miR-130a-3p was calculated using the 2^−ΔΔCt^ method.

### Statistical analysis

All the data were expressed as mean ± SD or number of cases. The differences between groups were analyzed using student’s t test (continuous variables) or Chi-square test (categorical variables). The risk factors of cerebral AS were screened using a logistic regression analysis, and the receiver operating characteristic (ROC) analysis was performed to evaluate the diagnostic performance of miR-130a-3p and the other screened risk factors. To explore the prognostic value of miR-130a-3p, its association with the 2-year cerebrovascular events of AS patients was assessed using Kaplan–Meier method and Cox regression analysis. To construct a prediction model of cerebrovascular events for AS patients, a logistic regression analysis was used to calculate the parameters in the risk model formula. All the statistical analyses were performed using SPSS software (SPSS, Inc., Chicago, USA) and GraphPad Prism software (Inc., Chicago, USA).

## Results

### Comparison of baseline characteristics of the study population

Cerebral AS patients contained 48 males and 26 females with an average age of 64.62 ± 8.69 years, and individuals in control group included 36 males and 26 females with an average age of 62.13 ± 8.34 years. There were no significant differences in age, gender, history of smoking and drinking, hypertension, diabetes, TG levels and HDL-C concentration between control and cerebral AS groups (all *P* > 0.05, Table 1). In patients with cerebral AS, the levels of hs-CRP, TC and LDL-C were significantly higher compared to the controls (all *P* < 0.001).

**Table 1:** Baseline characteristics of the participants

Characteristics	Controls (*n* = 62)	Cerebral AS (*n* = 74)	*P* value
Age (years)	62.13 ± 8.34	64.62 ± 8.69	0.092
Gender (male)	36	48	0.416
Smoking	26	37	0.348
Drinking	19	24	0.823
Hypertension	28	38	0.472
Diabetes	23	32	0.467
hs-CRP (mg/L)	9.54 ± 7.45	15.76 ± 11.68	< 0.001
TC (mM)	4.33 ± 0.80	5.37 ± 1.39	< 0.001
TG (mM)	1.52 ± 0.68	1.75 ± 0.73	0.064
LDL-C (mM)	2.53 ± 0.66	3.79 ± 0.65	< 0.001
HDL-C (mM)	1.20 ± 0.25	1.15 ± 0.17	0.153

*AS* Atherosclerosis, *hs-CRP* hypersensitivity C response protein, *TC* Total cholesterol, *TG* Triglyceride, *LDL-C* Low-density lipoprotein cholesterol, *HDL-C* High-density lipoprotein cholesterol

### Increased serum miR-130a-3p in patients with cerebral AS

**Fig. 1:**
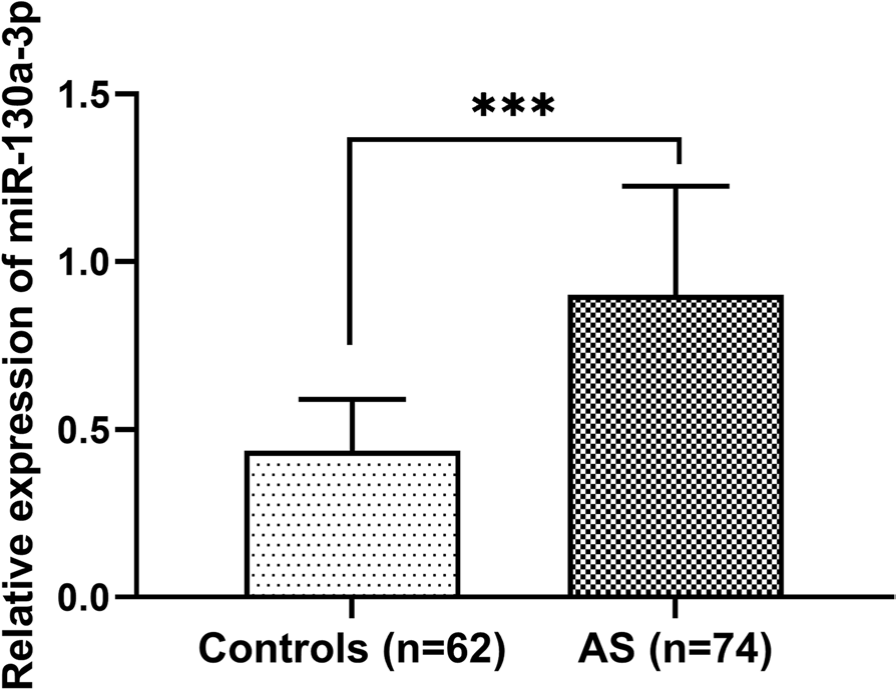
High expression of serum miR-130a-3p in cerebral AS patients compared with control individuals (***P < 0.001)

By examining the serum samples collected from the study population, we found that the expression of serum miR-130a-3p was markedly higher in AS patients than that in controls (*P* < 0.001, Fig. 1), suggesting the potential relationship of miR-130a-3p with AS development.

### Relationship analysis between serum miR-130a-3p with the clinical data of cerebral AS patients

To confirm the role of miR-130a-3p in the development of cerebral AS, its relationship with patients’ clinicopathological data was evaluated. To facilitate the relationship analysis, miR-130a-3p expression was firstly divided into low and high expression groups using its mean expression value (0.901). The results revealed that serum levels of miR-130a-3p were related with hypertension, TC and LDL-C levels (all *P* < 0.05), while it showed no significant relationship with patients’ age, gender, smoking, drinking, diabetes, hs-CRP, TG or HDL-C levels (all *P* > 0.05, Table 2).

**Table 2:** Association of miR-130a-3p with AS patients’ clinicopathological features

Variables	miR-130a-3p expression		*P* value
	Low (*n* = 35)	High (*n* = 39)	
Age (years)	62.86 ± 8.94	66.21 ± 8.25	0.098
Gender (male)	22	26	0.732
Smoking	15	22	0.244
Drinking	9	15	0.242
Hypertension	12	26	0.005
Diabetes	14	18	0.594
hs-CRP (mg/L)	15.65 ± 12.44	15.85 ± 11.11	0.942
TC (mM)	4.68 ± 1.19	5.99 ± 1.27	< 0.001
TG (mM)	1.71 ± 0.69	1.79 ± 0.78	0.614
LDL-C (mM)	3.42 ± 0.66	4.12 ± 0.43	< 0.001
HDL-C (mM)	1.17 ± 0.13	1.13 ± 0.20	0.241

*hs-CRP* hypersensitivity C response protein, *TC* Total cholesterol, *TG* Triglyceride, *LDL-C* Low-density lipoprotein cholesterol, *HDL-C* High-density lipoprotein cholesterol

### Risk factors of cerebral AS and their diagnostic value evaluation

This study used a logistic regression analysis to screen the potential risk factors of cerebral AS, and the results listed in Table 3 showed the relationship of hs-CRP, TC, TG, LDL-C, miR-130a-3p with the onset of cerebral AS (all *P* < 0.05). The further multivariate analysis results revealed that TC (OR = 1.686, 95% CI = 1.234–2.199, *P* = 0.039), LDL-C (OR = 2.003, 95% CI = 1.481–2.735, *P* = 0.036) and miR-130a-3p (OR = 2.202, 95% CI = 1.440–3.006, *P* = 0.012) were three risk factors of cerebral AS.

**Table 3:** Logistic analysis for the risk factors of cerebral AS

Variables	Univariate analysis		Multivariate analysis	
	OR (95% CI)	*P* value	OR (95% CI)	*P* value
Age	1.190 (0.708–1.781)	0.145	-	-
Gender	1.199 (0.686–1.945)	0.236	-	-
Smoking	1.236 (0.742–1.887)	0.184	-	-
Drinking	1.305 (0.628–2.038)	0.329	-	-
Hypertension	1.326 (0.891–1.911)	0.097	-	-
Diabetes	1.185 (0.704–1.733)	0.246	-	-
hs-CRP	1.267 (1.062–1.541)	0.045	1.223 (0.921–1.551)	0.091
TC	1.807 (1.293–2.438)	0.022	1.686 (1.234–2.199)	0.039
TG	1.137 (1.011–1.374)	0.041	1.114 (0.943–1.393)	0.072
LDL-C	2.052 (1.652–2.882)	0.015	2.003 (1.481–2.735)	0.036
HDL-C	0.882 (0.617–1.103)	0.094	-	-
miR-130a-3p	2.256 (1.543–3.104)	0.003	2.202 (1.440–3.006)	0.012

*hs-CRP* hypersensitivity C response protein, *TC* Total cholesterol, *TG* Triglyceride, *LDL-C* Low-density lipoprotein cholesterol, *HDL-C* High-density lipoprotein cholesterol

**Fig. 2:**
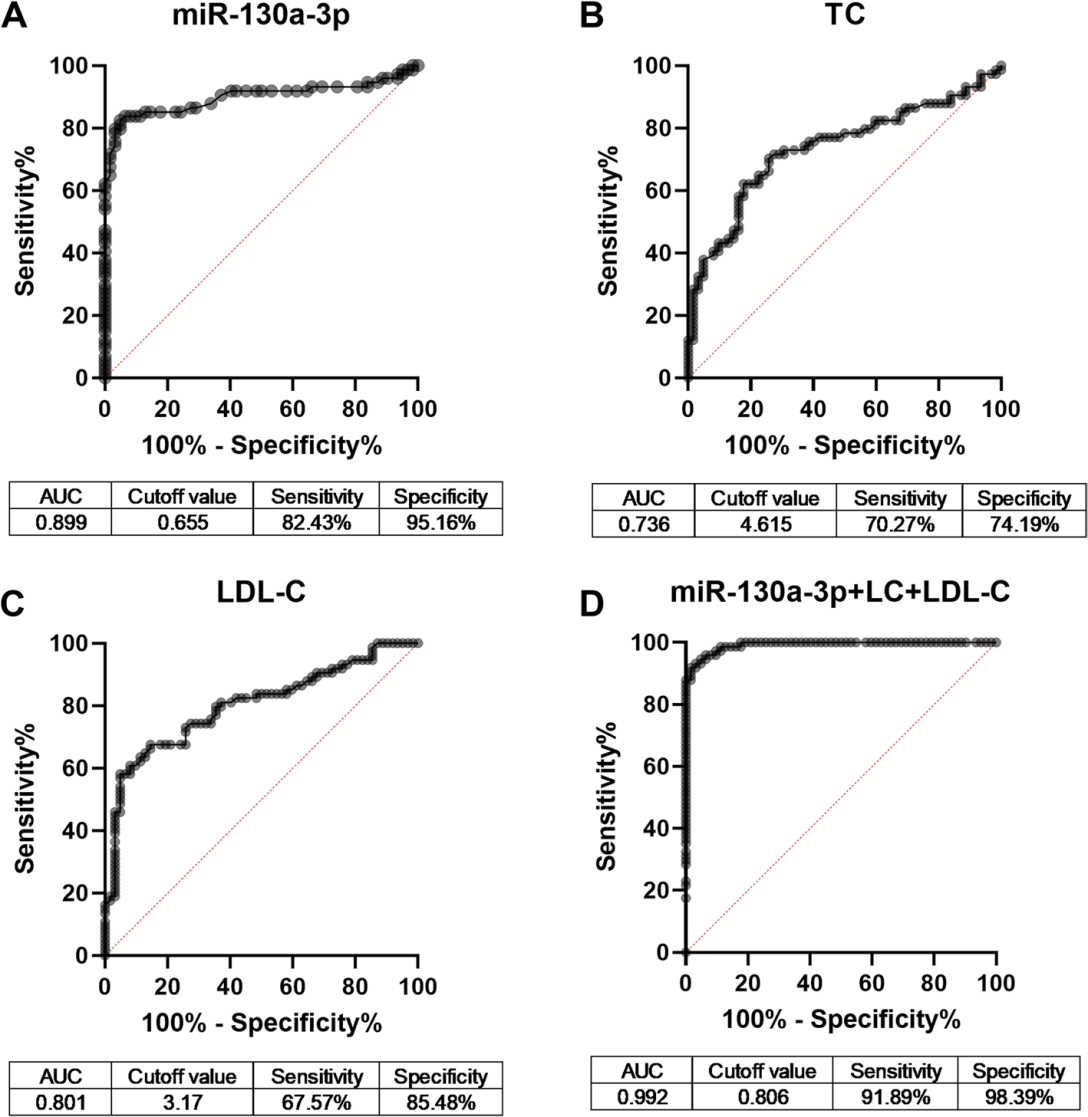
ROC curves based on miR-130a-3p, TC, LDL-C and their synthetic role in patients with cerebral AS patients. **A** ROC curve based on serum miR-130a-3p (AUC = 0.899). **B** ROC curve based on TC levels (AUC = 0.736). **C** ROC curve based on LDL-C levels (AUC = 0.801). **D** ROC curves based on the combination of miR-130a-3p, TC and LDL-C (AUC = 0.992). AUC: area under the curve

Furthermore, the screened risk factors were used to construct ROC curves to evaluate their diagnostic performance to distinguish AS cased from controls. The curves showed in Fig. 2 indicated that serum miR-130a-3p had relatively high diagnostic accuracy with an area under the curve (AUC) of 0.899 (cutoff value: 0.655; sensitivity: 82.43%; specificity: 95.16%) (Fig. 2A). In addition, the AUC values for TC and LDL-C were 0.736 and 0.801, respectively, with cutoff value of 4.615 for TC and 3.170 for LDL-C (Fig. 2B and C). More importantly, the joint diagnostic performance of miR-130a-3p, TC and LDL-C was remarkably improved than the three variables used alone, which had an AUC of 0.992, sensitivity of 91.89% and specificity of 98.39% (Fig. 2D).

### Circulating high miR-130a-3p independently predicts 2-year cerebrovascular events

**Fig. 3:**
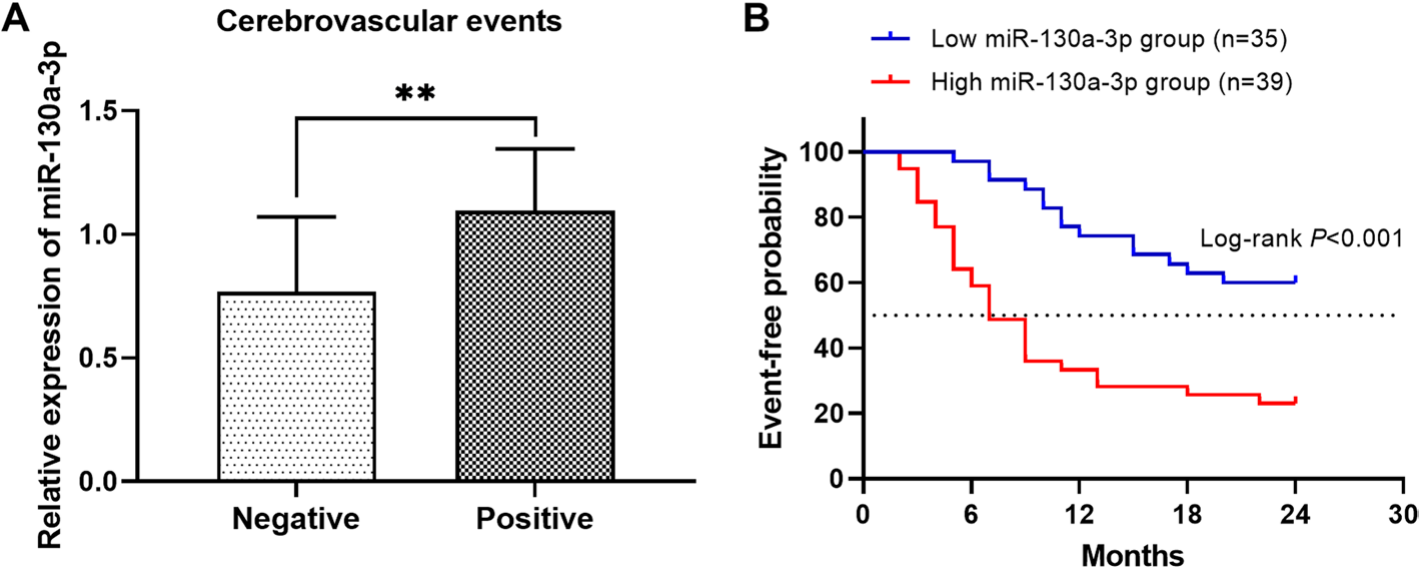
Relationship between miR-130a-3p and 2-year cerebrovascular events in cerebral AS patients. **A** Serum miR-130a-3p was elevated in patients with positive cerebrovascular events compared with those without adverse events (**P < 0.01). **B** High miR-130a-3p was associated with high probability of cerebrovascular events (log-rank *P* < 0.001)

Among the 74 cerebral AS patients, 44 (59.46%) cases developed cerebrovascular events during a 2 years follow-up survey. The patients with positive cerebrovascular events contained 30 (68.18%) TIA cases, 9 (20.46%) stroke cases and 5 (11.36%) deaths (died of cerebral infarction). The patients with cerebrovascular events had elevated serum miR-130a-3p compared with the cases without cerebrovascular events (*P* < 0.01, Fig. 3A). Additionally, a Kaplan–Meier method was used to draw cerebrovascular event-free curves to reflect the relationship between miR-130a-3p and event onset. The curves showed that high levels of serum miR-130a-3p were related with high probability of cerebrovascular events (log-rank *P* < 0.001, Fig. 3B). Subsequently, a Cox regression analysis was performed to check the independence of miR-130a-3p in the prediction of 2-year cerebrovascular events, and the results listed in Table 4 indicated that miR-130a-3p was an independent prognostic indicator for the occurrence of cerebrovascular events within 2 years (HR = 1.991, 95% CI = 1.202–3.298, *P* = 0.007). In addition, hs-CRP, TC, LDL-C and carotid stenosis were also independent factors for the prediction of 2-year cerebrovascular events (all *P* < 0.05).

**Table 4:** Cox regression analysis for 2-year cerebrovascular events

Variables	Univariate analysis		Multivariate analysis	
	HR (95% CI)	*P* value	HR (95% CI)	*P* value
Age	1.121 (0.863–1.445)	0.108	-	-
Gender	1.224 (0.735–1.589)	0.345	-	-
Smoking	1.238 (0.788–1.875)	0.271	-	-
Drinking	1.379 (0.903–1.901)	0.133	-	-
Hypertension	1.532 (0.935–2.183)	0.085	-	-
Diabetes	1.294 (0.918–1.778)	0.147	-	-
hs-CRP	1.204 (1.108–1.446)	0.033	1.173 (1.087–1.266)	0.042
TC	1.685 (1.226–2.114)	0.024	1.601 (1.210–2.118)	0.030
TG	1.331 (0.923–1.581)	0.076	-	-
LDL-C	1.943 (1.394–2.896)	0.019	1.737 (1.154–2.615)	0.024
HDL-C	0.803 (0.528–1.182)	0.088	-	-
Carotid stenosis	1.839 (1.126–2.915)	0.037	1.794 (1.113–2.892)	0.039
miR-130a-3p	2.117 (1.416–2.921)	0.002	1.991 (1.202–3.298)	0.007

*hs-CRP* hypersensitivity C response protein, *TC* Total cholesterol, *TG* Triglyceride, *LDL-C* Low-density lipoprotein cholesterol, *HDL-C* High-density lipoprotein cholesterol

### Construction of a logistic risk model to predict 2-year risk of cerebrovascular events

The parameters independently associated with 2-year cerebrovascular events, including miR-130a-3p, hs-CRP, TC and LDL-C, were analyzed using multivariate logistic regression analysis to obtain the coefficient of each parameter in a logistic regression equation (Table 5). Based on the equation *p* = Exp $\sum BiXi$/(1 + Exp $\sum BiXi$), a logistic risk model for 2-year cerebrovascular events was constructed with the following regression equation: *p*=1/[1+${\mathrm{e}}^{-(-2.110+0.643\times \mathrm{miR}-130\mathrm{a}-3\mathrm{p}+0.273\times \mathrm{hs}-\mathrm{CRP}+0.489\times \mathrm{TC}+0.517\times \mathrm{LDL}-\mathrm{C})}$].

**Table 5:** Multivariate logistic regression analysis results for 2-year cerebrovascular events

Variables	Estimated regression coefficient	OR (95% CI)	*P* value
miR-130a-3p	0.643	2.007 (1.222–2.901)	0.004
hs-CRP	0.273	1.181 (1.087–1.372)	0.041
TC	0.489	1.637 (1.214–2.101)	0.022
LDL-C	0.517	1.797 (1.182–2.482)	0.020

*hs-CRP* hypersensitivity C response protein, *TC* Total cholesterol, *LDL-C* low-density lipoprotein cholesterol

## Discussion

This study focused on the clinical significance of circulating miR-130a-3p in patients with cerebral AS, and a logistic risk model was finally constructed to predict 2-year cerebrovascular events in cerebral AS. Circulating expression of miR-130a-3p was found to be significantly upregulated in cerebral AS patients compared with controls, and was related with patients’ hypertension, TC and LDL-C levels. The risk factors of cerebral AS, including TC, LDL-C and miR-130a-3p, screened from this study had diagnostic accuracy to distinguish AS cases, and the joint detection of the three indicators had an improved diagnostic performance. Patients developed cerebrovascular events had higher miR-130a-3p expression than the cases without events, and high miR-130a-3p was independently associated with 2-year cerebrovascular events. A logistic risk model was constructed based on miR-130a-3p, hs-CRP, TC and LDL-C with the following regression equation: *p*=1/[1+${\mathrm{e}}^{-(-2.110+0.643\times \mathrm{miR}-130\mathrm{a}-3\mathrm{p}+0.273\times \mathrm{hs}-\mathrm{CRP}+0.489\times \mathrm{TC}+0.517\times \mathrm{LDL}-\mathrm{C})}$].

The pathogenesis of AS is complex involving the dysfunction of some types of cells, such as vascular smooth muscle cells and endothelial cells [15]. Thus, those molecules had regulatory effects on the function of these cells have received much attention in the development and progression of AS. For example, in an AS mice model, miR-377-3p could suppress vascular smooth muscle cell proliferation and migration, which was considered as a potential mechanism of miR-377-3p involving in AS pathogenesis [16]. As a functional miRNA, miR-130a-3p has also been reported to modulate the cell viability and migration of vascular smooth muscle cells [12, 13]. In our study, we found that serum miR-130a-3p was significantly elevated in cerebral AS patients compared with controls, indicating the potential role of miR-130a-3p in AS pathogenesis. In addition, miR-130a-3p was found to be associated with cerebral AS patients’ TC and LDL-C levels, indicating the potential relationship between miR-130a-3p and lipid metabolism. In thyroid eye disease, the elevated expression of miR-130a-3p was demonstrated to enhance lipid accumulation by inhibiting AMPK activity [17]. In addition to lipid metabolism, miR-130a-3p showed regulatory effects on inflammatory responses, which play critical roles in promoting AS development and progression [18]. Thus, miR-130a-3p might be involved in AS pathogenesis through influencing lipid metabolism and inflammation.

Accumulated evidence showed the important diagnostic potential of aberrant circulating miRNAs in various human diseases [19]. For the patients with AS, there are also some miRNAs with considerable diagnostic performance. The increased serum miR-186-5p in AS patients was identified as a potential diagnostic biomarker, and had promoting effects on vascular smooth muscle cell proliferation and migration [20]. Patients with AS-associated cerebra infarction had elevated serum miR-497 levels, which served as candidate biomarker for disease diagnosis and prognosis [21]. High levels of miR-130a-3p has been reported as a biomarker of AS obliterans [22], post intracerebral hemorrhage perihematomal edema [23] and bladder cancer [24]. In our study, the elevated serum miR-130a-3p was also found to have certain diagnostic accuracy to distinguish cerebral AS cases from controls, indicating its potential as a candidate diagnostic biomarker. In addition, TC and LDL-C levels are closely related with development and progression of AS, and have been identified as risk factors of AS, which was consistent with our analysis results [25, 26]. The ROC analysis of the present study indicated that the diagnostic potential of TC and LDL-C could be improved by miR-130a-3p, as evidenced by a high diagnostic accuracy by the combination of the three indicators.

As a pathological basis of TIA and stroke, AS needs to be concerned for its prognosis. The development of cerebrovascular events, especially the onset of stroke, significantly contributes to the disability and mortality of patients [27, 28]. During the 2-year follow-up survey in this study, 59.46% cerebral AS patients developed cerebrovascular events. These patients had significantly higher serum miR-130a-3p compared with those did not develop adverse events, and high miR-130a-3p was found to be independently associated with high probability of 2-year cerebrovascular events. In addition to miR-130a-3p, hs-CRP, TC and LDL-C were also identified as independent prognostic factors. Thus, the four prognostic factors were included into a logistic regression analysis, and a logistic risk model was constructed with the expectation to develop efficient prediction methods for the occurrence of cerebrovascular events in AS patients.

This study provide evidence for the clinical value of circulating miR-130a-3p in the diagnosis and prognosis of cerebral AS, but also included some limitations. The limited sample size is a major limitation, and future studies with larger study cohorts are necessary to confirm our conclusion. Second, the relationship analysis of laterality between stenosis and cerebrovascular events may provide novel and interesting results, however, the corresponding laterality information were not collected during the study. Third, we found the significant association of miR-130a-3p with TC and LDL-C levels of patients, but this study failed to further investigate the potential association between miR-130a-3p and lipid metabolism. Forth, previous studies demonstrated that miR-130a-3p had regulatory effects on the cell function of vascular smooth muscle cells, but this study did not perform mechanistic analysis. Thus, no cell experimental results were provided from our study to support the mechanism analysis. These are the limitations of this study, but it can also guide our future research.

In conclusion, serum miR-130a-3p was elevated in cerebral AS patients and related with patients’ hypertension condition, TC and LDL-C levels. Serum miR-130a-3p had considerable diagnostic potential for the screening of cerebral AS, and could improve the diagnostic accuracy of TC and LDL-C. In addition, increased miR-130a-3p predicted high probability of 2-year cerebrovascular events, and a logistic risk model based on serum miR-130a-3p, hs-CRP, TC and LDL-C might provide an efficient prediction method for the occurrence of cerebrovascular events in AS patients.

## Data Availability

The datasets generated and analyzed during the current study are not publicly available due [CONFIDENTIALITY] but are available from the corresponding author on reasonable request.

## Abbreviations

AS: Cerebral atherosclerosis
miR-130a-3p: MicroRNA-130a-3p
TC: Total cholesterol
LDL-C: Low-density lipoprotein cholesterol
AS: Atherosclerosis
miRNAs: MicroRNAs
UTR: 3'-Untranslated region
RT-qPCR: Reverse transcription quantitative PCR
